# Causal associations between gut microbiota and rheumatoid arthritis: A two-sample Mendelian randomization study

**DOI:** 10.1097/MD.0000000000042596

**Published:** 2025-05-30

**Authors:** Feiran Wei, Meng Zhao, Xiaohui Sun, Hongfei Ma, Huimin Yin, Xiaobing Shen

**Affiliations:** a Key Laboratory of Environmental Medicine Engineering, Ministry of Education, School of Public Health, Southeast University, Nanjing, Jiangsu, China; b Department of Epidemiology and Health Statistics, School of Public Health, Southeast University, Nanjing, Jiangsu, China; c Medical Affairs Department, Qingdao Hongdao People’s Hospital No. 330, Qingdao, Shandong, China.

**Keywords:** causal association, gut microbiota, Mendelian randomization, rheumatoid arthritis

## Abstract

The gut microbiota has been implicated in the development of rheumatoid arthritis (RA), but whether these associations reflect causal relationships remains unclear. We conducted a two-sample Mendelian randomization analysis to investigate the potential causal effects of gut microbial taxa on RA risk. Summary-level data from the MiBioGen consortium (n = 13,266) and a large RA genome-wide association study (n = 97,173) were used. Multiple Mendelian randomization methods and sensitivity analyses were applied to ensure robustness. Four microbial taxa showed nominal associations with RA. Increased abundance of Catenibacterium, Desulfovibrio, and Ruminiclostridium 6 was associated with a higher risk of RA, while Lachnospiraceae (UCG008) appeared to have a protective effect. Although these associations did not meet Bonferroni-corrected significance, results were consistent across analytical methods with no evidence of pleiotropy or heterogeneity. This study provides genetic evidence supporting a potential causal link between specific gut microbes and RA risk. The findings highlight host immune modulation as a possible pathway connecting the gut microbiome to RA and identify candidate taxa for future mechanistic and therapeutic research.

## 1. Introduction

Rheumatoid arthritis (RA) is a chronic, systemic, inflammatory, destructive autoimmune disease, and affecting ~1% of the world population.^[1]^ It is characterized by hyperplastic synovium, persistent synovitis, injuries of joints, and articular cartilage, etc.^[2]^ RA causes overall damage to the organism, which limits patient mobility, affects daily life, and reduces overall quality of life.^[3]^ The exact mechanisms underlying the onset and progression of RA are unknown, but there is no doubt that it is influenced by genetic and environmental factors. In addition, a growing number of studies have demonstrated that RA is frequently associated with an imbalance of gut microflora.^[4]^

The human gut microbiota (GM) consists of a wide variety of bacteria that play a vital role in maintaining overall health.^[5]^ Research has shown that the GM has been linked to a wide range of diseases, including psychiatric disorders,^[6]^ hematological disorders^[7]^, and autoimmune disorders.^[8]^ Currently, many large-scale studies have shown that a portion of the bacterial taxa in the gut have genetic effects.^[9,10]^ It has been shown that there may be a correlation between the GM and RA.^[11,12]^ However, confounding factors (environmental, lifestyle, and dietary habits) often influence traditional observational studies and greatly interfere with inferences about risk factor causality in relation to endpoint outcomes. In addition, establishing causal relationships will not only deepen GM’s understanding of autoimmune disorders pathogenesis, but also have the ability to guide clinical programs targeting microbiota as an intervention-directed therapy. Therefore, there is an urgent need to elucidate the causal relationship between GM and RA.

Mendelian randomization (MR), which controls for confounders and reduces bias, uses single nucleotide polymorphisms (SNPs) as instrumental variables (IVs) to infer causal correlations between exposures and outcomes.^[13]^ MR is extensively used in order to understand the causal relationship between GM and disease.^[14]^ MR studies have been analyzed and discussed regarding the causal association between GM and RA, but the results have been inconsistent. In this study, we performed MR analyses using the latest genome-wide association studies (GWAS) data to assess the causal association between GM and RA.

## 2. Materials and methods

### 2.1. Ethics

This study used publicly available GWAS summary statistics and required no ethical approval.

### 2.2. Study’s design and assumptions

**Figure 1.**
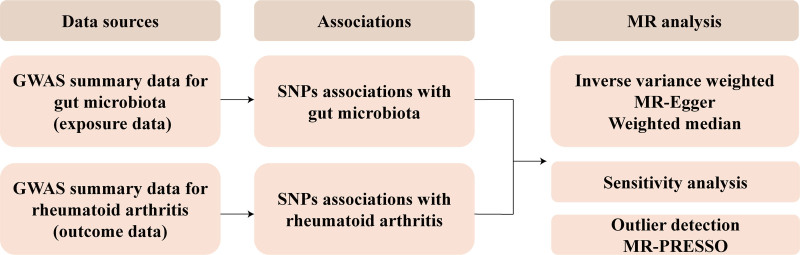
dThe workflow of the study.

**Figure 2.**
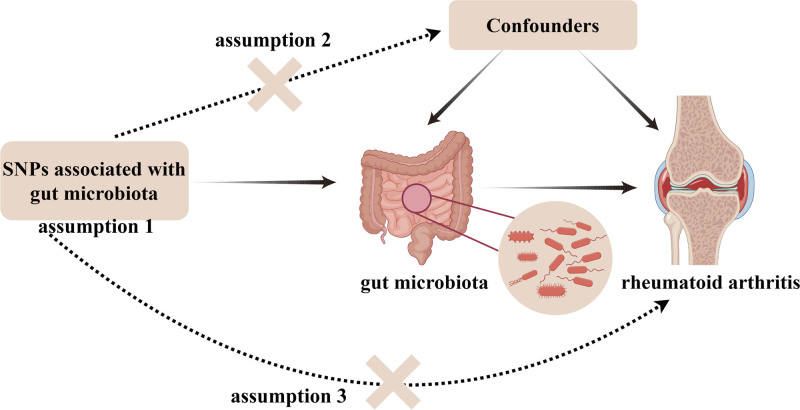
The assumptions of MR. MR = Mendelian randomization.

Using STROBE-MR guidelines,^[15]^ this study was designed. A brief description of the MR design is displayed in Figure 1. In short, GM served as the exposure and RA served as the outcome. After certain conditions for screening, SNPs significantly associated with GM were selected as IVs. Meanwhile, 3 assumptions must be met for IVs (Fig. 2): (1) Relevance (the IVs are highly correlated with the exposure); (2) Independence (the IVs are not associated with confounders); and (3) Exclusion restriction (IVs should influence the outcome only through the exposure).

### 2.3. Data sources

Genetic information on GM was acquired from a large GWAS conducted by the MiBioGen (https://mibiogen.gcc.rug.nl/) consortium including 18,340 participants (~78% European ancestry).^[16]^ The MiBioGen consortium keeps data from the largest genome-wide meta-analysis to date on GM composition.^[17]^ In this database, 131 genera with a mean abundance >1% were found, including 12 unknown genera at the lowest taxonomic level.^[16]^ As a result, we analyzed 119 genus-taxa in the present study. We obtained the latest data for RA from the GWAS catalog (https://www.ebi.ac.uk/gwas).^[18]^ This data included 35,871 RA patients and 240,149 controls from 37 cohorts, from which we selected the European population (22,350 cases and 74,823 controls) as the target data.

### 2.4. Selection of genetic variants as IVs

To ensure the robustness of the data and the accuracy of the results, we select IVs through strict criteria. First, SNPs closely associated with GM classification were selected as IVs (*P* < 1.0 × 10^‐5^)^[19]^; Second, SNPs associated with RA were excluded (*P* < 1 × 10^−5^); Third, linkage disequilibrium between SNPs was calculated by using the 1000 Genomes Project’s European samples data to ensure independence between SNPs (*r*^2^ < 0.001, clumping distance = 10,000 kb); Fourth, to avoid weak instrument bias, SNPs with an *F*-statistic (*F* = Beta^2^/Se^2^) below 10 were included in the analysis.^[20]^

### 2.5. Statistical analysis and sensitivity analysis

MR analysis was performed to assess the causal association between GM and RA. Inverse variance weighted (IVW)^[21]^ was the primary analysis method, while MR-Egger^[22]^ and weighted median^[23]^ were complementary methods. In order to more accurately assess the causal associations obtained through MR analyses, this study determined *P*-value (*P* < .05/119 = .000420) using Bonferroni multiple tests correction.^[24]^ Also, a sensitivity analysis was conducted in this study. First, the horizontal pleiotropy of IVs was assessed using the MR-PRESSO global test^[25]^ and MR-Egger regression^[22]^; Second, Cochran *Q* test^[26]^ was used to assess heterogeneity among IVs; Third, leave-one-out sensitivity analysis^[27]^ was used to assess the effects of a single SNP. Statistical analysis was performed using R (version 4.3.1) software utilizing the R package “TwoSampleMR” (version 0.5.6). GM taxa are considered causally related to RA when the following conditions are met^[28]^: (1) IVW method results were statistically different; (2) The estimation results of the 3 MR methods (IVW, MR-Egger, and weighted median) are in the same direction; and (3) the results of Cochran *Q* test, MR-Egger test, and MR-PRESSO global test were not statistically significant (*P* > .05). It is worth noting that a nominal/potential causal effect was considered to be present if the *P*-value was <.05 but above the Bonferroni correction threshold^[29]^.

### 2.6. Mapping SNPs to genes

The SNP annotation was carried out using FUMA^[30]^ v1.6.0 (https://fuma.ctglab.nl). In FUMA’s “SNP2GENE” tool, we used the expression quantitative trait locus strategy to identify genes associated with SNPs used in the MR analysis.

### 2.7. Pathway and functional enrichment analyses

The functional annotation was performed using the Gene Ontology (GO) (https://geneontology.org) and Kyoto Encyclopedia of Genes and Genomes (KEGG) (https://www.genome.jp/kegg/) databases.^[31]^ A significant KEGG pathway and GO term enrichment was determined by applying the hypergeometric test and considering *P* < .05 as the threshold.

## 3. Results

### 3.1. Causal effects

**Figure 3.**
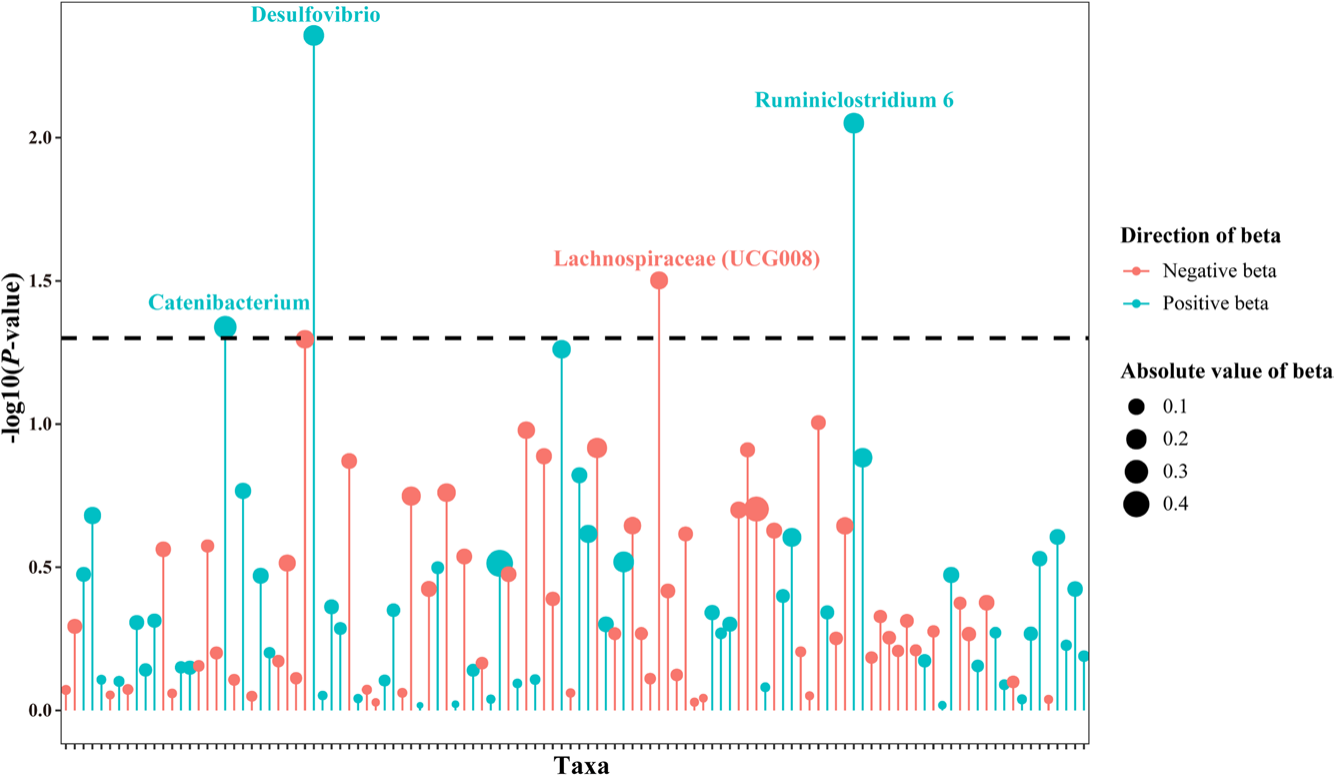
Lollipop plot of 119 IVW-results of GM on RA. IVW = inverse variance weighting, GM = gut microbiota, RA = rheumatoid arthritis.

**Figure 4.**
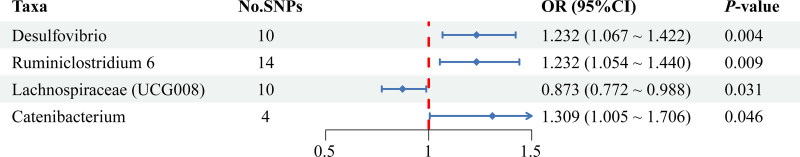
Forest plot of IVW analysis results of the effect of GM on RA. IVW = inverse variance weighting, GM = gut microbiota, RA = rheumatoid arthritis.

The results of the MR analysis of 119 Taxa on RA are exhibited in Figure 3. Further, the statistical results are presented in Figure 4 and Figure S1, Supplemental Digital Content, https://links.lww.com/MD/P69. It showed that genetically predicted Catenibacterium (OR = 1.309, 95% CI: 1.005–1.706, *P* = .046), Desulfovibrio (OR = 1.232, 95% CI: 1.067–1.422, *P* = .004), and Ruminiclostridium 6 (OR = 1.232, 95% CI: 1.054–1.440, *P* = .009) were positively correlated with the risk of RA, suggesting that the above-mentioned taxa had adverse effect on RA. In addition, genetically predicted Lachnospiraceae (UCG008) (OR = 0.873, 95% CI: 0.772–0.988, *P* = .031) was negatively related to the risk of RA, suggesting the protective effect on RA (Table S1, Supplemental Digital Content, https://links.lww.com/MD/P69). However, there was no statistically significant difference in the results after the Bonferroni correction.

The significant taxa’s SNPs are shown in Table S2, Supplemental Digital Content, https://links.lww.com/MD/P69. In addition, they were less likely to suffer weak instrument bias in the research since the *F*-statistics for all selected SNPs were larger than 10.

### 3.2. Sensitivity analysis

Using a sensitivity analysis, we evaluated the robustness of the results. Cochran *Q* test (Table S4, Supplemental Digital Content, https://links.lww.com/MD/P69) and leave-one-out analysis (Figure S2, Supplemental Digital Content, https://links.lww.com/MD/P69) indicated no heterogeneity among the SNPs.

### 3.3. Mapping SNPs to genes

**Figure 5.**
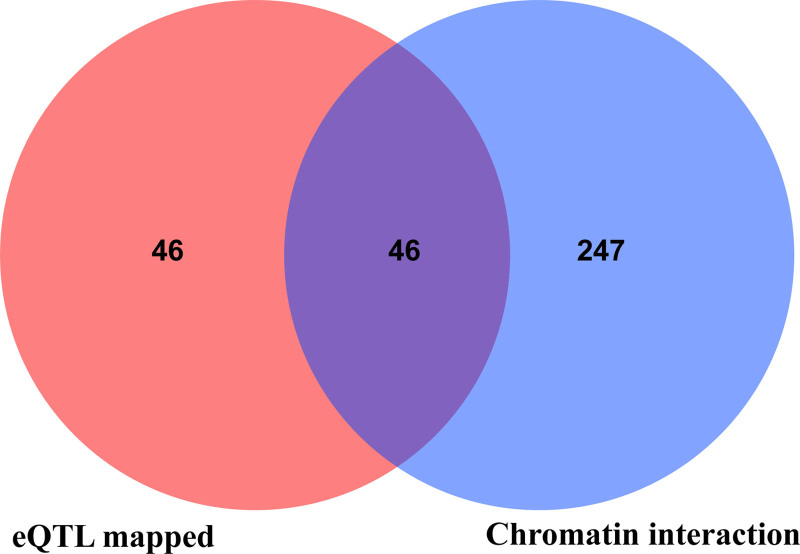
Genes mapped to GM-related SNPs. GM = gut microbiota, SNPs = single nucleotide polymorphisms.

We identified and annotated the SNPs used in the MR analysis by 2 gene mapping methods. The expression quantitative trait locus-based method resulted in 92 genes; the chromosome interaction-based method resulted in 293 genes (Fig. 5 and Table S3, Supplemental Digital Content, https://links.lww.com/MD/P69).

### 3.4. Gene set enrichment analyses

**Figure 6.**
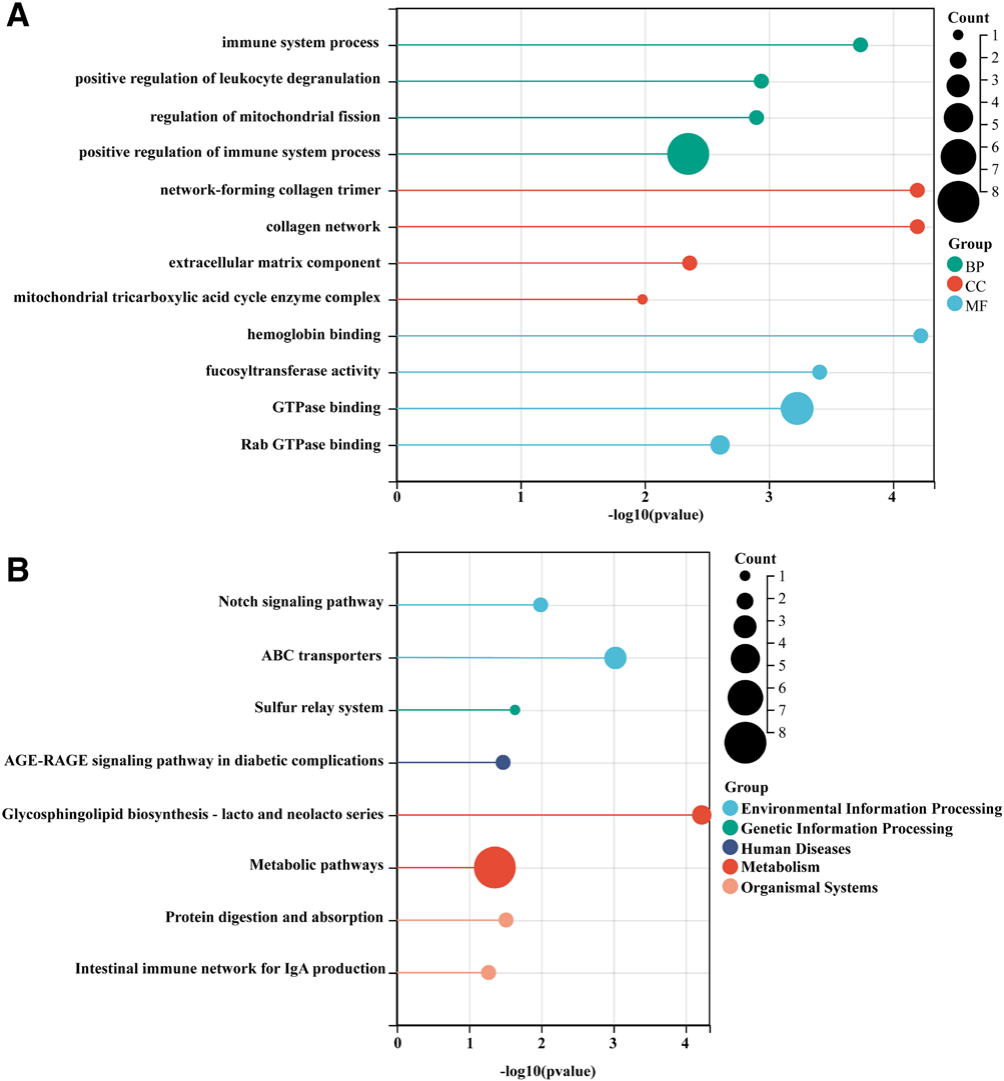
Enrichment analysis. (A) GO; (B) KEGG.

To explore the possible mechanisms by which GM causes RA, 46 genes were identified in this study, and GO enrichment analyses were performed to understand the relevant functions of the genes. In addition, gene signaling pathway analysis was performed using the KEGG database to understand the signaling pathways in which the genes are involved. In terms of biological process, genes are mainly involved in the immune system process, positive regulation of leukocyte degranulation, regulation of mitochondrial fission, and positive regulation of immune system process. In terms of cell component, genes are mainly concentrated in network-forming collagen trimer, collagen network, extracellular matrix component, and mitochondrial tricarboxylic acid cycle enzyme complex, etc. In terms of molecular function, these genes were mainly related to hemoglobin binding, fucosyltransferase activity, GTPase binding, and Rab GTPase binding. KEGG enrichment pathway analysis showed that these genes were involved in environmental information processing, genetic information processing, human diseases, metabolism, and organismal systems (Fig. 6).

## 4. Discussion

In this study, we conducted a two-sample MR analysis to explore the potential causal relationship between GM and RA, using the largest GM GWAS data from the MiBioGen consortium and the most up-to-date RA GWAS summary statistics. This study revealed that Catenibacterium, Desulfovibrio, and Ruminiclostridium 6 may be positively associated with increased RA risk, while Lachnospiraceae (UCG008) may have a protective effect.

Although none of the taxa remained statistically significant after Bonferroni correction, this method is known to be overly conservative, especially when tests are correlated, as is often the case in microbiome studies.^[32–34]^ To better balance type I and type II errors, we also applied the Benjamini–Hochberg false discovery rate (FDR) correction. While no associations passed the FDR < 0.05 threshold, Desulfovibrio and Ruminiclostridium 6 showed suggestive associations (FDR ≈ 0.053). These results are not statistically conclusive and are presented as exploratory findings to inform future research.

The GM play a key role in regulating immune functions and metabolism in the host.^[35]^ In addition, GM is associated with the onset and development of chronic inflammatory diseases and metabolic disorders in the body.^[36]^ Research suggests that dysregulated gut ecology may contribute to the development or further deterioration of autoimmune diseases including RA, ankylosing spondylitis, systemic lupus erythematosus.^[37,38]^ A correlation between GM and RA has now been established through observational studies.^[39]^ Previous studies have shown that the diversity of gut microbial species is significantly altered in RA patients. However, previous studies have been observational and have their inherent limitations, making it difficult to determine the causal association between GM and RA.

Desulfovibrio is the most abundant genus of commensal sulfate-reducing bacteria in the human colon.^[40]^ It has been shown to stimulate epithelial immune responses, leading to the production of inflammatory cytokines by macrophages.^[41]^ Fu et al conducted a cross-sectional analysis using a multiracial cohort and found that a significant positive correlation between the relative abundance of genus Desulfovibrio and inflammatory factor levels in the general population.^[42]^ Similarly, an experimental animal study showed a strong correlation between Desulfovibrio and systemic chronic inflammation in the body.^[43]^ On the one hand, many strains in this genus are opportunistic pathogens that are associated with a number of inflammatory diseases; on the other hand, members of Desulfovibrio produce endotoxins.^[44]^ This implies that Desulfovibrio may play a pathogenic role in RA.

The specific functions and roles of Lachnospiraceae (UCG008) are not fully understood. It is worth noting that the Lachnospiraceae family are anaerobic, spore-forming bacteria that degrade complex polysaccharides into short chain fatty acids.^[45]^ It has been shown that many short chain fatty acids-producing bacteria (including Lachnoclostridium, Lachnospira, and Sutterella) are significantly reduced in patients with systemic autoimmune diseases, and that these bacteria have a strong pro-regulatory effect on immune processes.^[46]^ A cohort study showed that Lachnospiraceae was significantly less abundant in patients with autoimmune diseases (multiple sclerosis, inflammatory bowel disease, and rheumatoid arthritis).^[47]^ Meanwhile, Liu et al published an article showing reduced abundance of Lachnospiraceae in RA rats.^[8]^ All of the above clues support the results of our MR analyses that Lachnospiraceae is a protective factor for the development of RA.

Our findings differ from some previous MR studies, which identified different RA-associated microbial taxa. For instance, Gou et al^[48]^ and Zhou et al^[49]^ reported associations with taxa such as Bacteroidaceae, Desulfovibrionales, and Ruminococcaceae UCG013. In contrast, our study highlighted Desulfovibrio, Ruminiclostridium 6, and Catenibacterium as potential risk factors. These differences may result from variations in datasets and methods, and reflect the complexity of microbiota–RA interactions.

Notably, we found for the first time that increased abundance of Catenibacterium and Ruminiclostridium 6 can increase the risk of RA. Catenibacterium is a Gram-positive anaerobic bacterium. Several studies reported an increase in the abundance of Catenibacterium in infectious and autoimmune disorders,^[50]^ suggesting its association with a pro-inflammatory response. Ruminiclostridium is a strictly anaerobic bacterium.^[51]^ An animal study showed that the relative abundance of Ruminiclostridium 6 was positively correlated with levels of the pro-inflammatory factors IL-17A, TNF-α, and lipopolysaccharide, inferring that Ruminiclostridium 6 may promote inflammation in vivo.^[52]^

To further explore the potential mechanisms underlying these associations, we performed gene set enrichment analysis based on SNPs mapped to host genes associated with microbial abundance. Previous literature has proposed that gut microbes may influence RA through immune modulation.^[53,54]^ The “gut-joint axis” model suggests that dysbiosis-induced microbial products may translocate to the synovium via immune cells, contributing to joint inflammation. Moreover, the GM has been implicated in the pathogenesis of other systemic diseases, such as cardiovascular disorders, via similar immune-mediated pathways,^[55]^ reinforcing the importance of the gut-immune axis.

Our enrichment results support the immune modulation hypothesis. The mapped genes were significantly enriched in immune-related pathways, such as leukocyte activation and cytokine signaling. Importantly, genes such as *IL7*, *TAGAP*, and *DHODH*—which play established roles in T-cell development, immune signaling, and pharmacological targeting in RA—were among those mapped. These data support a genetically mediated link between host immunity and GM composition.^[56,57]^ In contrast, more speculative mechanisms—such as microbial translocation or antigen mimicry^[58,59]^—were not directly evaluated in this study and remain topics for future research.

## 5. Limitations

First, none of the microbial associations remained statistically significant after applying Bonferroni or FDR correction, which may reflect limited statistical power for some taxa and the conservative nature of multiple testing adjustments in microbiome data. Nevertheless, some taxa showed nominal significance (*P* < .05), and these findings are presented as exploratory and require validation in independent datasets and experimental models.

Second, the GWAS summary statistics used were predominantly derived from individuals of European ancestry. Although principal component analysis was applied to adjust for population structure, residual stratification bias cannot be fully excluded. Moreover, due to the use of summary-level data, ancestry-specific analyses could not be performed. As a result, the generalizability of our findings to non-European populations remains uncertain and warrants further validation in more diverse cohorts.

Third, the lack of individual-level data precluded stratification of RA cases by clinical characteristics such as disease stage, severity, serological status, or treatment response. Therefore, we could not assess whether the observed associations differ across RA subtypes. Future studies with access to individual-level clinical data would be valuable in evaluating whether these associations vary by RA subtype or disease stage.

Despite these limitations, our study provides a novel perspective on the gut-joint axis by integrating genetic instruments with microbial data, laying a foundation for future mechanistic and clinical investigations.

## 6. Conclusions

In conclusion, this study provides genetic evidence supporting a potential causal relationship between specific gut microbial taxa and the risk of rheumatoid arthritis. Our findings suggest that host immune modulation may be a key pathway linking gut microbiota to RA pathogenesis. While these results offer novel insights into the gut-joint axis, further studies in diverse populations and experimental models are needed to validate these associations and clarify the underlying biological mechanisms. This work may inform future strategies for microbiome-based prevention or treatment of RA.

## Acknowledgments

We want to acknowledge the participants and investigators of the FinnGen study and GWAS catalog.

## Author contributions

**Conceptualization:** Feiran Wei, Meng Zhao.

**Data curation:** Feiran Wei, Meng Zhao.

**Formal analysis:** Feiran Wei, Meng Zhao, Xiaohui Sun.

**Investigation:** Hongfei Ma.

**Methodology:** Meng Zhao, Huimin Yin.

**Project administration:** Hongfei Ma.

**Resources:** Hongfei Ma.

**Supervision:** Xiaobing Shen.

**Writing – original draft:** Meng Zhao.

**Writing – review & editing:** Meng Zhao.

## Supplementary Material


